# Radioiodine Refractory Follicular Thyroid Cancer and Surgery for Cervical Relapse

**DOI:** 10.3390/cancers13246230

**Published:** 2021-12-11

**Authors:** Costanza Chiapponi, Milan J. M. Hartmann, Matthias Schmidt, Michael Faust, Anne M. Schultheis, Christiane J. Bruns, Hakan Alakus

**Affiliations:** 1 Department of General, Visceral, Cancer and Transplant Surgery, University Hospital of Cologne, Kerpener Str. 62, 50937 Cologne, Germany; milan.hartmann@smail.uni-koeln.de (M.J.M.H.); christiane.bruns@uk-koeln.de (C.J.B.); hakan.alakus@uk-koeln.de (H.A.); 2 Department of Nuclear Medicine, Faculty of Medicine, University Hospital of Cologne, Kerpener Str. 62, 50937 Cologne, Germany; matthias.schmidt@uk-koeln.de; 3 Polyclinic for Endocrinology, Diabetes and Preventive Medicine, University Hospital of Cologne, Kerpener Str. 62, 50937 Cologne, Germany; michael.faust@uk-koeln.de; 4 Department of Pathology, University Hospital of Cologne, Kerpener Str. 62, 50937 Cologne, Germany; anne.schultheis@uk-koeln.de

**Keywords:** follicular thyroid cancer, radioiodine therapy, radioiodine refractory thyroid cancer, metastatic follicular thyroid cancer

## Abstract

**Simple Summary:**

Differentiated thyroid cancer includes papillary (PTC) and follicular thyroid cancer (FTC). Eighty-five percent of patients are cured by thyroidectomy and one single radioiodine treatment. Relapse is rare and can mostly be cured by a second radioiodine therapy. Between 5% and 10% of differentiated thyroid cancers are, or become, unresponsive to radioiodine treatment during the course of disease. For these patients repeated surgery is the only curative option: chemotherapy and external beam radiation play a minor role, targeted therapy is started in a palliative setting for rapidly progressing tumors. In most studies on radioiodine refractory differentiated thyroid cancer, there is a predominance of PTC, due to its higher incidence compared to FTC. In the present study we critically evaluate indications and outcome of repetitive cervical surgery for radioiodine refractory FTC at our university clinic.

**Abstract:**

Compared to its more common counterpart papillary thyroid cancer (PTC), follicular thyroid cancer (FTC) has a less favorable outcome, due to its higher incidence of distant metastases and advanced stages at diagnosis. Despite radioiodine (RAI) avidity, metastatic FTC often progresses after radioiodine treatment (RAIT). We aimed at evaluating the indications and outcomes of surgery for cervical relapse of radioiodine refractory FTC. Patients receiving RAIT between 2005 and 2015 at the University Hospital of Cologne, Germany, were screened. Patients with FTC were identified. Demographics, clinic-pathologic characteristics, treatment, and outcome of patients diagnosed with RAI refractory FTC, who underwent cervical surgery in the course of disease, were analyzed. FTC accounted for 8.8% of all thyroid carcinomas undergoing RAIT. In 35.2% of FTC patients, disease persisted or recurred despite a cumulative mean RAI activity of 18.7 GBq ± 11.6 (follow-up 83.5 ± 56.7 months). Distant metastases were diagnosed in 75% of these patients, as bone (57.6%), lung (54.6%), and liver metastases (12.1%). Cervical relapse occurred in 63.6% of these patients and was treated in 57.1% with surgery with, and without, external beam radiation therapy (EBRT). Despite surgery and EBRT, in 75% of patients, cervical relapse recurred again. In conclusion, surgery for cervical radioiodine refractory FTC relapse is often performed in metastatic setting. With and without EBRT, cure is rare, although metastases can appear radioiodine avid. Early biological marker and systemic treatments for these patients are still needed.

## 1. Introduction

Follicular thyroid cancer (FTC) is the second most common differentiated thyroid cancer histological type. It has been described to account for 4% to 39% of all thyroid cancers, with higher incidence in the presence of iodine deficiency and [1] no association with radiation [2]. Compared to its more common counterpart, papillary thyroid cancer (PTC), it has a less favorable outcome [3,4] due to its higher incidence of distant metastasis and the higher amount of patients presenting with advanced stage disease [2,5]. Distant metastases have been described to be present and/or develop in 6 to 20% of FTC patients [2], as bone metastases in 42%, lung metastases in 33%, and lymph node metastases in 8% of patients [6].

In a retrospective analysis of patients with metastatic differentiated thyroid cancer (DTC) submitted to radioiodine therapy (RAIT) including 17 FTC patients, Simoes-Pereira et al. [7] described that radioiodine (RAI) avidity was highest in follicular variant PTC and FTC compared to classic PTC and hurtle cell carcinoma. RAI avidity has also been described elsewhere to correlate with RAS mutations as opposed to BRAF mutations, which are more associated with RAI unresponsiveness [8,9]. RAS mutations have been reported in 30–40% of FTCs [10,11] in contrast to BRAF mutations, which are not displayed in FTC [10,12]. Despite higher RAI avidity, FTC histology resulted in significantly more often progression after RAIT and worse progression free survival (PFS) compared to classic PTC [7]. This discrepancy between initial RAI avidity and progression after RAIT is contemplated as category 3 in the ETA Guidelines for the management of radioiodine refractory thyroid cancer, described as “progression despite uptake of RAI” [13].

Despite some unique aspects and some differences from PTC, there are no specific recommendations for radioiodine refractory FTC [3]: FTC is mostly included in the wider category DTC, which contains tumors carrying different genomic alterations and displaying some different clinical behaviors, also found in the radioiodine unresponsive version. The lack of RAI avidity in diagnostic whole-body scintigraphy scan (DWBS) alongside with 18F-FDG avidity, for example, are a useful element for prediction of poor radioiodine response in radioiodine refractory PTC [14], FTC in contrast can display higher RAI avidity in DWBS but then progress after RAIT, nonetheless.

In the present study, we critically evaluate the clinical course of patients with invasive FTC receiving RAIT at our institution, with focus on repeated cervical surgery indicated by the multidisciplinary endocrine team (MDET) in case of 18F-FDG-PET positive loco regional FTC relapse. Demographics, clinic-pathologic characteristics, oncologic treatment, and outcome of these cases were analyzed, in order to find out whether cervical surgery had any impact on the outcome of radioiodine refractory FTC and whether there were any specific differences in comparison with radioiodine refractory PTC.

## 2. Materials and Methods

### 2.1. Patients

Patients receiving RAIT between 1 January 2005 and 31 December 2015 at the University Hospital of Cologne, Germany, for thyroid cancer were screened. Primary surgical treatment had been often performed in peripheral hospitals and these patients had been referred for radioiodine treatment and oncologic care with thyroid carcinoma diagnosis to our center. The subgroup of patients with FTC was identified. The German Guidelines [15] recommend total thyroidectomy followed by ablative radioiodine treatment for patients with macroinvasive (widespread infiltration of thyroid tissue and/or blood vessel) FTC. Minimally invasive FTC (MIFTC) without vascular invasion is deemed as cured by hemithyroidectomy.

Demographics, clinic-pathologic characteristics, oncologic treatment, and outcome were analyzed.

### 2.2. Diagnosis of Recurrence

Diagnosis of recurrence was based on physical examination, thyroglobulin (Tg) increment and imaging studies. During follow-up, significant elevation of basal and stimulated serum TG compared to the nadir value and in general > 1 ng/mL measured with an ultrasensitive assay led to the administration of 185–370 MBq of radioiodine (I-131) to obtain a diagnostic whole-body scintigraphy scan (DWBS) and—in case of low or absent radioiodine uptake—to an ^18^F-FDG PET-CT. If DWBS studies were positive, patients received a therapeutic activity of 3.7–7.4 GBq of radioiodine (I-131). If DWBS was negative, but ^18^F-FDG PET-CT confirmed structural recurrence, RAI response was considered to be low and local treatments including surgery were discussed. RAIT was generally not repeated in patients with significantly progressing disease after RAIT, according to the ETA Guidelines for the management of radioiodine refractory thyroid cancer [8].

If the imaging studies did not display recurrence, serum Tg levels were monitored, as described before [9].

### 2.3. Surgery for Recurrence

**Figure 1:**
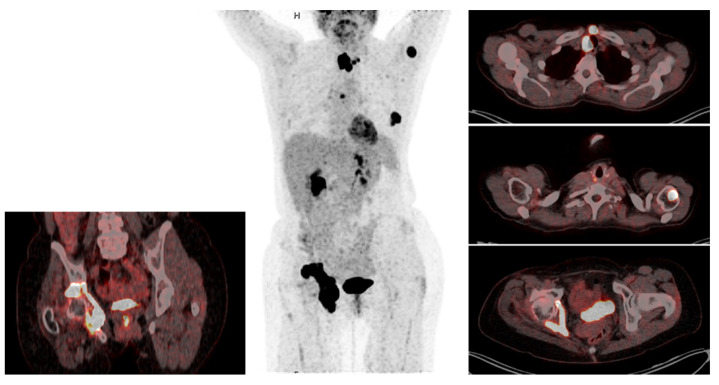
Cervical surgery in the setting of metastatic disease. This 55-year-old female patient underwent R0 resection of a minimally invasive pT3 cN0 cM0 FTC with vascular invasion 2014 and received her first RAIT. She first presented at our university clinic two years later, when she was diagnosed with cervical recurrence. She underwent cervical surgery, followed by cervical EBRT for diffuse soft tissue micrometastatic disease. Three years later, she was diagnosed with a new cervical relapse despite EBRT and a pelvic metastasis, which was deemed not resectable by orthopedic surgery. After her third cervical surgery in 2019, revealing soft tissue metastatic disease, she received a second RAIT and pelvic EBRT. These are the 18F-FDG PET scan and die DWBS six years after begin of disease. In addition to the pelvic metastasis and a further cervical relapse, new bone metastases are visible. The patient underwent her fourth cervical surgery this year. The trachea resulted superficially infiltrated over several cm, making a tracheal resection in the setting of metastatic disease not reasonable. A shaving resection was performed. Lenvatinib, which had been started 2020, was not tolerated well by the patient and treatment was discontinued. Disease is currently slowly progressing.

Surgery was generally indicated if DWBS was negative, but 18F-FDG PET-CT confirmed structural recurrence. Additionally, in case of radioiodine uptake but large tumor burden, pretherapeutic surgery was advocated in order to debulk tumor mass and reduce the burden to clear for radioiodine treatment or EBRT. Mostly surgical treatment consisted of cervical re-exploration and resection of the local recurrence. Indication to surgery was always initiated by the multidisciplinary endocrine team (MDET). In contrast to radioiodine refractory PTC [16], surgery for radioiodine refractory 18F-FDG-PET avid loco cervical recurrence of FTC was generally performed in patients displaying bone and/or other distant metastases (Figure 1).

### 2.4. EBRT

External Beam Radiation Therapy (EBRT) of the neck was individually advised in case of histologically proven incomplete tumor clearance for treating micro- and sometimes macroscopic residual disease, after thorough information concerning the poor current evidence and the possible side effects of treatment [13,17]. Treatment decisions were individualized taking into account patients’ age, personal preferences, and potential side effects. Cervical EBRT was performed either in palliative (50 Gy) or in curative intention (59.4–63 Gy) over 8 weeks. Bone metastases, in contrast, including sternum metastases, were regularly treated with EBRT. This treatment however was not object of this study.

### 2.5. Follow-Up

Follow-up examinations take place yearly in the department for Nuclear Medicine and include physical examinations, thyroglobulin level, DWBS, cervical ultrasound, and/or MRI and ^18^FDG PET-CT, if necessary. Mean follow-up was 83.5 ± 56.7 months after last thyroidectomy. Follow-up examinations until September 2021 were included in this study. Response was evaluated according to the RECIST criteria.

### 2.6. Data Collection and Analysis

Electronic and paper data of the University Hospital of Cologne were collected and analyzed. The study was approved by the ethical committee of the University Hospital Cologne (ID: 21-1541). Data were analyzed using IBM SPSS Statistics for Windows, Version 25.0. Armonk, NY, USA.

## 3. Results

### 3.1. Patient Characteristics

A total of 1414 patients with thyroid cancer received postoperative radioiodine therapy in our department of nuclear medicine; 125 (8.8%) patients had FTC. The average age of these FTC patients was 53 ± 16.4 years without any difference between genders. Three (2.3%) patients had minimally invasive FTCs (MIFTC) and received RAIT according to the MDET because of reported R1 resection [18]. Subtotal resections were performed in ten (8%) patients, two-stage total thyroidectomy in fifty-six (44.8%) patients, and primary total thyroidectomy in fifty-nine (47.2%) patients.

### 3.2. Tumor Stages and Metastatic Disease at the Time of Diagnosis (TNM)

A total of 34 (27.2%) of 125 patients had a pT1b-, 46 (36.8%) a pT2-, 30 (24%) a pT3-, and 8 (6.4%) a pT4-status. In seven (5.6%) cases T status was not documented.

In 60 (48%) patients lymph nodes were additionally resected according to the judgment of the operating surgeon at the time of first surgery. Lymph nodal metastases (pN+) were diagnosed in 12 (20%) patients (4 men, 8 female; average age 61.5 ± 17.2 years). The status of the primary tumor for patients with lymph nodal metastasis was pT1b in two (16.6%) cases, pT3 in four (33.3%) cases, and pT4 in two (16.6%) cases. In four (33.3%) cases the T status was not documented. Four (33.3%) patients with lymph node metastasis also had distant metastasis (cM1) at the time of diagnosis while a further four (33.3%) patients developed distant metastasis during the course of their disease.

Distant metastasis (cM1) was seen in 15 (12%) patients (average age 61.8 ± 14.3 years) at the time of diagnosis, including metastasis to bones in 6 (40%), to the lung in 8 (53.4%) and to the liver in 2 (12.5%) patients. One patient had both bone and liver metastases at diagnosis. Patients with distant metastases were mostly females (*n* = 13, 86.6%). They had one (6.7%) pT1-, one (6.7%) pT2-, seven (46.6%) pT3-, and three (20%) pT4-status. Two (13.3%) patients had widely invasive tumors with widespread infiltration of thyroid tissue. In three (23%) cases, the thyroid gland had been removed years before and histology had been classified as benign, FTC diagnosis was made by metastases biopsy.

Patients with lymph nodal and/or metastatic disease (*n* = 23) were in average significantly older than those without (61.7± 15 versus 52 ± 16.2 years, *p* = 0.0067).

### 3.3. Treatment—RAIT

FTC patients received RAIT with an activity of either 3.7 (*n* = 80, 64%) or 5.5 GBq (*n* = 5, 4%). In the presence of lymph node and/or distant metastases at first diagnosis (*n* = 23, 18.4%) they received 7.4 GBq. In 17 (13.6%) cases the first radioiodine treatment had not been performed at our institution and the activity administered was not documented. Tg normalized in 64.8% (*n* = 81) of patients after RAIT.

A total of 37 (29.6%) patients received a RAIT cumulative mean activity > 7.4 GBq (24.1± 14 GBq in average) due to biochemical and/or structural disease persistence or progression. In only three (2.45) of these patients, Tg normalized.

### 3.4. Treatment—Surgery for Cervical Relapse

Loco regional radioiodine refractory 18F-FDG-PET positive recurrence was diagnosed in 21 (16.8%) of all FTC patients with a predominance in women (71.4%). These patients had received a treatment with a cumulative mean activity of 19.1 ± 16.1 GBq. The average age of these patients was 62.7 years ± 11.

The primary tumor status included one (4.7%) pT1, six (28.6%) pT2, eight (38%) pT3, five (23.8%) pT4, and in one case, pT-status was not documented. In 8 (38%) cases they also displayed bone metastases, in 13 (61.9%) lung, and in 3 (14.1%) liver metastases.

Repeated cervical surgery was recommended by the MDET and performed in 12 (57.1%) of these 21 patients. Table 1 summarizes their characteristics, treatment, and outcome (Table 1).

Table 1: characteristics, tumor stadium, additional metastases, treatment, FU, and outcome of patients who underwent repeated cervical surgery for radioiodine refractory FTC at our institution between 2005 and 2015. Three (25%) patients were male; there were no pT1 tumors but four (33.3%) pT2, five (41.6%) pT3, and three (25%) pT4. All but three patients (75%) had distant metastases. The cumulative mean activity received by these patients was 21.2 ± 18.7 GBq, seven of them received adjuvant external beam radiation. Only one patient (8.3%) had neither biochemical nor structural disease (remission = R) in a follow-up of 135 months. There was one (8.3%) incomplete response (=I.R.) with elevated Tg without structural disease, two (16.6%) stable disease (=S.D.), whereas all other patients showed slow progress (P) during last follow-up. Patient 4 was lost to follow-up after 6 months after he was diagnosed with progressing disease. “E” = exitus letalis, “n.d.” non documented.

Nine (75%) women and three (25%) male patients underwent cervical surgery for radioiodine refractory FTC disease. Their initial tumor stages included no pT1 tumors but four (33.3%) pT2, five (41.6%) pT3, and three (25%) pT4 tumors. All but three patients (75%) had distant metastases at the time of repeated cervical surgery. The cumulative mean activity received by these patients previously to repeated cervical surgery was 21.2 ± 18.7 GBq.

In seven (58.3) cases; adjuvant external beam radiation was recommended by the MDET and performed after cervical surgery.

### 3.5. Overall Oncologic Outcome of FTC

A total of 81 (64.8%) patients undergoing radioiodine treatment for FTC were cured by thyroidectomy and RAIT, in one case after additional resection of a cervical relapse. Three (3.7%) patients are currently free of biochemical and structural disease, despite metastatic stage at the time of diagnosis. One 32-year-old patient had isolated lung metastases (FU 164 months, last check 03/2019). Two patients (32 and 47 years old) had synchronous lung and bone metastases at the time of diagnosis (FU 111 and 137 months, last check 02 and 03/2021, respectively). In all three cases, one RAIT was sufficient to achieve cure.

In 44 (35.2%) patients cure could not be achieved with thyroidectomy and RAIT; although, these patients received a cumulative mean activity of 18.7 GBq ± 11.6 (3.7–55.3 GBq).

In 11 (25%) cases a biochemical incomplete response without structural disease was diagnosed at last follow-up.

In 33 (75%) patients, structural metastatic disease, consisting of 18F-FDG-PET positive foci was diagnosed. Bone metastases were present in 19 (57.6%) patients. Six (31.6%) had them at the time of diagnosis, whereas thirteen (68.4%) developed them in average 26.1 months after FTC diagnosis. Eighteen (54.5%) had lung metastases either since diagnosis (*n* = 8, 44.4%) or in average 28.8 months after diagnosis (*n* = 10, 55.6%). Two had (11.1%) and two (50%) developed liver metastases (*n* = 4, 12.1%) during the first 26 months after diagnosis. Two (11.1%) patients with both lung and bone metastases, also developed brain metastases.

These 33 patients were significantly older than those achieving cure (63 ± 10 versus 47.5 ± 16, *p* = 0.0001), had more advanced pT status (3% pT1, 21% pT2, 36.4% pT3, 21.2% pT4, 18.2% n.d. versus 39.6% pT1, 41.9% pT2, 18.5% pT3, 0% pT4; *p* = 0.0003), and similar gender distribution (male 30% versus 33.7%).

In five (15.5%) cases Sorafenib treatment was started but had to be discontinued because of side effects. In one (3%) case, displaying 43% RAS mutation redifferentiation with trametinib was attempted, without significant response though. Besides five (15.5%) patients who died, the other 84.8% were slowly progredient and managed conservatively (symptom control) at last the follow-up (mean FU 93.8 ± 51.7).

### 3.6. Oncologic Outcome after Surgery for Cervical FTC Relapse

Surgery for cervical FTC relapse was performed in 12 patients (Table 1). Only one (8.3%) patient has currently neither biochemical nor structural disease (R in Table 1) in a follow-up of 135 months.

**Figure 2:**
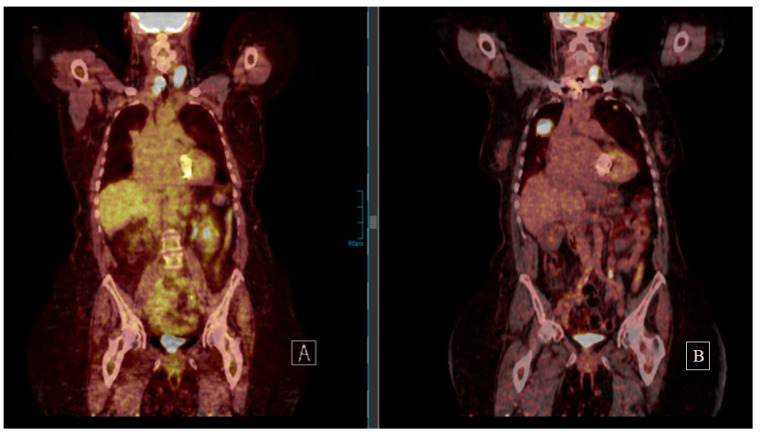
Patient 5 underwent resection of a cervical FTC recurrence diagnosed 5 years after thyroidectomy despite bilateral lung metastases (18F-FDG PET on the left side (**A**)). Cervical and thorax EBRT were performed. The 18F-FDG PET on the right (**B**) (5 years after the second cervical resection and 10 years after first FTC diagnosis) shows both cervical and lung progress. After thorough patient information concerning the evidence on tyrosine kinase inhibitors, the patient refused treatment and was progressing at last follow-up.

There is one (8.3%) incomplete response (I.R.) with elevated Tg without structural disease, two (16.6%) stable disease (S.D.) cases, whereas all other patients showed slow progress during last follow-up. Patient 4 was lost to follow-up after 6 months. Among the eight (66.7%) patients receiving both surgery and EBRT, only patient 6 and 8 had no further cervical recurrence after the repeated surgical resection. In the other six (75%) cases, the cervical relapse recurred again despite surgery and EBRT (Figure 2).

## 4. Discussion

In the present study we concentrate on advanced, radioiodine refractory follicular thyroid cancer (FTC) for finding out, in which cases repeated cervical surgery was indicated and whether it positively influenced the outcome of patients. We also aimed at finding out possible differences to PTC, since FTC and PTC are commonly discussed together as “radioiodine refractory” DTC.

The first observation is that FTC was the reason of radioiodine treatment in only 8.8% of patients at our institution. This is consistent with most literature reporting FTC to account for 4% to 39% of all thyroid cancers [2]. For this reason, only 125 FTCs were identified in a period of 11 years. The high incidence of distant metastasis and patients presenting with more advanced stage disease was confirmed by the data presented, in which distant metastases were found in 12% of patients at the time of diagnosis, consistently with the previous literature [2,3]. The higher rate of distant metastases occurring during the course of disease reported in this study (35% of patients as opposed to 6 to 20% [2]) is probably because FTCs undergoing radioiodine treatment, as included in this study, are usually high-risk tumors (angioinvasive and widely invasive variants). Minimally invasive FTCs without vascular invasions are usually treated by surgery only, according to the German Guidelines [15]. Only three of these patients received RAIT and were included, being diagnosed with R1 resection. Since many patients were referred to our university center after primary surgery elsewhere for radioiodine treatment, a certain patients’ selection must be taken into account.

The second observation is that cervical surgery for radioiodine refractory FTC relapse was performed rarely (12 patients over 11 years) as compared to PTC (30 patients over 10 years, [16]). However, the percentage of patients undergoing repeated cervical surgery for radioiodine refractory FTC over all FTCs is higher than those undergoing surgery for radioiodine refractory PTC over all PTCs (9.6% of all FTC patients in contrast to 3% of all PTC patients [16], *p* = 0.0001). Surgical resection of radioiodine refractory cervical FTC recurrence was generally performed in the setting of metastatic disease (75% of cases) and in significantly older patients compared to PTC patients (62.6 ± 12.9 versus 44.6 ± 17 [16], *p* < 0.002). These two aspects explain why the remission rate in patients undergoing repeated cervical surgery for radioiodine refractory FTC is very low (one patient free of disease, two in stable disease, 58.3% of patients experiencing slow progress and two dead). Concerning remission rates though, no comparison with our previously presented collective [16] can be made, because it included isolated cervical PTC relapses undergoing salvage surgery in the presence of no or only few lung metastases.

Another aspect to be mentioned is the striking heterogeneity of FTC response to RAIT, whereas those FTCs, who achieved cure, required one radioiodine treatment with an activity of 3.7–7.4 GBq after thyroidectomy, and metastatic FTC received generally several radioiodine treatments and a cumulative activity of 18.7 GBq ± 11.6 (3.7–55.3 GBq) in average. In fact, FTC metastatic foci are typically RAI avid in DWBS [7], but more often significantly progress after RAIT [7], resulting in a higher propensity to treatment as opposed to radioiodine refractory PTC. In the study of Parameswaran et al. [6], however, there was no statistical difference in survival for patients with RAIT-avid metastases who received RAIT versus those who did not. Although their collective included only 16 patients, this study is one of few considering FTC specifically instead of FTC alongside with other types of differentiated cancers. More FTC specific data appear to be needed.

Our data confirm the impact of age as prognostic factor [13,17,19,20]: patients who could be cured in this study were in average 47.5 ± 16 years old, and thus significantly younger than those in whom cure could not be achieved (65 ± 9.7 years, *p* < 0.0001). Three young patients (32, 32, and 47 years old) could be cured by one surgical procedure and RAIT even despite lung and bone metastases (Tg < 0.2 and no evidence of disease in a follow-up of FU 111, 164, and 137 months, respectively).

Finally, the role of adjuvant cervical EBRT remains controversial [21,22,23,24,25,26,27]. Although this study includes the course of seven patients receiving adjuvant EBRT, only two (28.6%) of them took benefit of treatment in terms of cervical clearance of disease. Of course, EBRT is generally recommended for R1 resections and more often in elderly patients, in order to avoid further cervical surgery, causing a certain patients’ selection. Considering that EBRT makes further surgical treatment challenging, more evidence appears to be needed in order to figure out if, and which, patients are candidates for cervical adjuvant EBRT, given the lack of effective systemic adjuvant options [28,29].

We conclude that radioiodine refractory FTC was performed rarely and in the setting of metastatic disease in older patients as opposed to salvage surgery for radioiodine refractory PTC. Patients had received RAIT with higher cumulative mean activity (21.2 ± 18.7 GBq versus 10.5 ± 6.2 GBq, *p* = 0.014), which is probably due to the more aggressive nature of FTC but also to the discrepancy between RAI avidity of FTC metastases and progression despite RAIT.

Despite the relatively small patients’ collective and the retrospective nature of this analysis our data strongly suggest that cervical surgery has the important role of reducing/delaying local complication, facilitating RAIT effect in case of high tumor burden, but cannot achieve neither cure nor cervical tumor clearance in most cases. Its outcome can probably rarely be enhanced by EBRT. More prospective and FTC specific data are required.

## 5. Conclusions

Radioiodine refractory cervical FTC recurrence is resected more often than radioiodine refractory PTC in metastatic setting for symptom palliation and/or for delaying local complications. Cervical relapse is frequent also after cervical EBRT. More data are needed as to the role of EBRT. Biomarkers for recognizing which metastatic FTC might benefit of repeated RAIT and repeated cervical surgery should be auspicated in the future.

## Figures and Tables

**Table 1:** Characteristics, treatment, and outcome of patients undergoing repeated cervical surgery for loco regional radioiodine refractory recurrence.

	Gender	Age (Years)	Initial TNM	Additional Metastases at the Time of Cervical Surgery	Cumulative Mean RAIT Activity	EBRT (Gy)	FU (Months)	Outcome
Pt1	m	58	pT3 pN0(0/12) cM0	lung	14.8		143	S.D.
Pt2	w	77	pT4 pN1 cM0	lung, skin	55.3	60	121	P
Pt3	w	29	pT2 pNx pM1	lung, liver	11.1	50.4	129	P
Pt4	w	67	pT4 pNx cM0	lung	29.6		6	P
Pt5	w	65	pT3 pN0(0/26) cM1	lung	12.9	n.d.	114	P
Pt6	m	55	pT2 pNx cM0		3.7	60	135	R
Pt7	w	68	pT3 pNx cM0	lung	48.6		133	S.D.
Pt8	w	73	pT2 pNx cM0		11.1	60	53	I.R.
Pt9	m	57	pT3 pN1 (3/12) cM0		3.7		19	E
Pt10	w	59	pT2 pNx cM0	bone, brain	42.4	60	116	E
Pt11	w	74	pT3 pN0(0/26) cM1	lung	9.2	60	68	P
Pt12	w	69	pT4a pN1 (19/52) cM0	lung, bone, liver	12.9	59.4	71	P

## Data Availability

Data can be required on reasonable request to the author any time.

## References

[1] Huszno B., Szybiński Z., Przybylik-Mazurek E., Stachura J., Trofimiuk-Müldner M., Buziak-Bereza M., Gołkowski F., Pantoflinski J. (2003). Influence of Iodine Deficiency and Iodine Prophylaxis on Thyroid Cancer Histotypes and Incidence in Endemic Goiter Area. J. Endocrinol. Investig..

[2] Ashorobi D., Lopez P.P. Follicular Thyroid Cancer. https://www.ncbi.nlm.nih.gov/books/NBK539775/.

[3] Grani G., Lamartina L., Durante C., Filetti S., Cooper D.S. (2018). Follicular Thyroid Cancer and Hürthle Cell Carcinoma: Challenges in Diagnosis, Treatment, and Clinical Management. Lancet Diabetes Endocrinol..

[4] Passler C., Scheuba C., Prager G., Kaczirek K., Kaserer K., Zettinig G., Niederle B. (2004). Prognostic Factors of Papillary and Follicular Thyroid Cancer: Differences in an Iodine-Replete Endemic Goiter Region. Endocr. Relat. Cancer.

[5] Iwasaki H., Toda S., Murayama D., Kato S., Matsui A. (2021). Surgical Indications and Clinical Management of Benign and Malignant Follicular Thyroid Tumors: An Algorithmic-Based Approach. Mol. Clin. Oncol..

[6] Parameswaran R., Shulin Hu J., Min En N., Tan W.B., Yuan N.K. (2017). Patterns of Metastasis in Follicular Thyroid Carcinoma and the Difference between Early and Delayed Presentation. Ann. R. Coll. Surg. Engl..

[7] Simões-Pereira J., Mourinho N., Ferreira T.C., Limbert E., Cavaco B.M., Leite V. (2021). Avidity and Outcomes of Radioiodine Therapy for Distant Metastasis of Distinct Types of Differentiated Thyroid Cancer. J. Clin. Endocrinol. Metab..

[8] Agrawal N., Akbani R., Aksoy B.A., Ally A., Arachchi H., Asa S.L., Auman J.T., Balasundaram M., Balu S., Baylin S.B. (2014). Integrated Genomic Characterization of Papillary Thyroid Carcinoma. Cell.

[9] Sabra M.M., Dominguez J.M., Grewal R.K., Larson S.M., Ghossein R.A., Tuttle R.M., Fagin J.A. (2013). Clinical Outcomes and Molecular Profile of Differentiated Thyroid Cancers With Radioiodine-Avid Distant Metastases. J. Clin. Endocrinol. Metab..

[10] Theurer S., Rawitzer J., Ting S., Schmid K.W. (2021). Diagnostic Principles of Thyroid Tumors in Pathology: Relevant Changes Due to the Current WHO Classification. Pathologe.

[11] Song Y.S., Lim J.A., Min H.S., Kim M.J., Choi H.S., Cho S.W., Moon J.H., Yi K.H., Park D.J., Cho B.Y. (2017). Changes in the Clinicopathological Characteristics and Genetic Alterations of Follicular Thyroid Cancer. Eur. J. Endocrinol..

[12] Kebebew E., Weng J., Bauer J., Ranvier G., Clark O.H., Duh Q.Y., Shibru D., Bastian B., Griffin A. (2007). The Prevalence and Prognostic Value of BRAF Mutation in Thyroid Cancer. Ann. Surg..

[13] Fugazzola L., Elisei R., Fuhrer D., Jarzab B., Leboulleux S., Newbold K., Smit J. (2019). 2019 European Thyroid Association Guidelines for the Treatment and Follow-Up of Advanced Radioiodine-Refractory Thyroid Cancer. Eur. Thyroid J..

[14] Kang S.Y., Bang J.-I., Kang K.W., Lee H., Chung J.-K. (2019). FDG PET/CT for the Early Prediction of RAI Therapy Response in Patients with Metastatic Differentiated Thyroid Carcinoma. PLoS ONE.

[15] German Association of General and Visceral surgery (2012). Operative Therapy of Malignant Thyroid Cancer. https://www.dgav.de/fileadmin/media/texte_pdf/caek/Leitlinie_Maligne_Schilddruesenerkrankungen_Operative_Therapie_2012-11.pdf.

[16] Chiapponi C., Alakus H., Faust M., Schultheis A.M., Rosenbrock J., Schmidt M. (2021). Salvage Surgery for Cervical Radioiodine Refractory 18F-FDG-PET Positive Recurrence of Papillary Thyroid Cancer. Endocr. Connect..

[17] Haugen B., Alexander E., Bible K.C., Doherty D., Mandel S., Nikiforov Y.E., Pacini F., GW R., Sawka A.M., Schlumberger M. (2016). 2015 American Thyroid Association Management Guidelines for Adult Patients with Thyroid Nodules and Differentiated Thyroid Cancer: The American Thyroid Association Guidelines Task Force on Thyroid Nodules and Differentiated Thyroid Cancer. Thyroid.

[18] Ban E.J., Andrabi A., Grodski S., Yeung M., Mclean C., Serpell J. (2012). Follicular Thyroid Cancer: Minimally Invasive Tumours Can Give Rise to Metastases. ANZ J. Surg..

[19] Machens A., Lorenz K., Weber F., Dralle H. (2021). Risk Patterns of Distant Metastases in Follicular, Papillary and Medullary Thyroid Cancer. Horm. Metab. Res..

[20] Sugino K., Kameyama K., Nagahama M., Kitagawa W., Shibuya H., Ohkuwa K., Uruno T., Akaishi J., Suzuki A., Masaki C. (2014). Follicular Thyroid Carcinoma with Distant Metastasis: Outcome and Prognostic Factor. Endocr. J..

[21] Hamilton S.N., Tran E., Berthelet E., Wu J. (2017). The Role of External Beam Radiation Therapy in Well-Differentiated Thyroid Cancer. Expert Rev. Anticancer. Ther..

[22] Chen P.V., Ahn E., Avitia S., Osborne R., Juillard G. (2002). Adjuvant External Beam Radiotherapy in High Risk Well-Differentiated Thyroid Cancer. Int. J. Radiat. Oncol. Biol. Phys..

[23] So K., Smith R.E., Davis S.R. (2016). Radiotherapy in Well-Differentiated Thyroid Cancer: Is It Underutilized?. ANZ J. Surg..

[24] Yang Z., Flores J., Katz S., Nathan C.A., Mehta V. (2017). Comparison of Survival Outcomes Following Postsurgical Radioactive Iodine Versus External Beam Radiation in Stage IV Differentiated Thyroid Carcinoma. Thyroid.

[25] Megwalu U.C., Orloff L.A., Ma Y. (2019). Adjuvant External Beam Radiotherapy for Locally Invasive Papillary Thyroid Cancer. Head Neck.

[26] Giuliani M., Brierley J. (2014). Indications for the Use of External Beam Radiation in Thyroid Cancer. Curr. Opin. Oncol..

[27] Carrillo J.F., Flores J.M., Espinoza G., Vázquez-Romo R., Ramírez-Ortega M.C., Carrillo L.C., Cortés-García B.Y., Ochoa-Carrillo F.J., Oñate-Ocaña L.F. (2021). Treatment of Unresectable Differentiated Thyroid Carcinoma With Upfront External Radiotherapy and Salvage Surgery: A STROBE-Compliant Retrospective Cohort Study. Front. Oncol..

[28] Lamartina L., Godbert Y., Nascimento C., Do Cao C., Hescot S., Borget I., Al Ghuzlan A., Hartl D., Hadoux J., Pottier E. (2020). Locally Unresectable Differentiated Thyroid Cancer: Outcomes and Perspectives. Endocrine.

[29] Fussey J.M., Crunkhorn R., Tedla M., Weickert M.O., Mehanna H. (2016). External Beam Radiotherapy in Differentiated Thyroid Carcinoma: A Systematic Review. Head Neck.

